# Genomic characterization of Ugandan smallholder farmer‐preferred cassava varieties

**DOI:** 10.1002/csc2.20152

**Published:** 2020-05-04

**Authors:** Paula Iragaba, Robert S. Kawuki, Guillaume Bauchet, Punna Ramu, Hale A. Tufan, Elizabeth D. Earle, Michael A. Gore, Marnin Wolfe

**Affiliations:** ^1^ Plant Breeding and Genetics Section, School of Integrative Plant Science Cornell Univ. Ithaca NY 14853 USA; ^2^ National Crops Resources Research Institute (NaCRRI) PO Box 7084 Kampala Uganda; ^3^ Boyce Thompson Institute Cornell Univ. 533 Tower Rd. Ithaca NY 14853 USA; ^4^ Cornell University, Institute for Genomic Diversity 175 Biotechnology Building Ithaca NY 14853 USA; ^5^ International Programs/College of Agriculture and Life Science B75 Mann Library Ithaca NY 14853 USA

## Abstract

Understanding the genetic relationships among farmer‐preferred cassava (*Manihot esculenta* Crantz) varieties is indispensable to genetic improvement efforts. In this study, we present a genetic analysis of 547 samples of cassava grown by 192 smallholder farmers, which were sampled at random within four districts in Uganda. We genotyped these samples at 287,952 single nucleotide polymorphisms using genotyping‐by‐sequencing and co‐analyzed them with 349 cassava samples from the national breeding program in Uganda. The samples collected from smallholders consisted of 86 genetically unique varieties, as assessed using a genetic distance‐based approach. Of these varieties, most were cultivated in only one district (30 in Kibaale, 19 in Masindi, 14 in Arua, and three in Apac), and only three were cultivated across all districts. The genetic differentiation we observed among farming districts in Uganda (mean fixation index [*F*_ST_] = .003) is similar to divergence observed within other countries. Despite the fact that none of the breeding lines were directly observed in farmer fields, genetic divergence between the populations was low (*F*_ST_ = .020). Interestingly, we detected the presence of introgressions from the wild relative *M. glaziovii* Müll. Arg. on chromosomes 1 and 4, which implies ancestry with cassava breeding lines. Given the apparently similar pool of alleles in the breeding germplasm, it is likely that breeders have the raw genetic material they require to match the farmer‐preferred trait combinations necessary for adoption. Our study highlights the importance of understanding the genetic makeup of cassava currently grown by smallholder farmers and relative to that of plant breeding germplasm.

**Abbreviations**
GBS: genotyping‐by‐sequencing
IBS: identity‐by‐state
IRB: Institutional Review Board
LD: linkage disequilibrium
MAF: minor allele frequency
NaCRRI: National Crops Resources Research Institute
PC: principal component
PCA: principal component analysis
SNP: single nucleotide polymorphism

## 1 INTRODUCTION

Cassava (*Manihot esculenta* Crantz) is an important source of food to ∼800 million people globally (FAO, 2013). Although cassava was originally domesticated in Latin America (Allem, 1999), it is currently grown all over the tropics at latitudes between 30° N and 30° S (Ceballos, Iglesias, Perez, & Dixon, 2004). More than half of the total cassava produced in the world is grown in sub‐Saharan Africa (FAO, 2016), where it ranks as the second most important staple food crop (Nweke, Spencer, & Lynam, 2002). Cassava is cultivated by smallholder farmers as a reliable source of food and income crop in unstable environments: it is vegetatively propagated and tolerates marginal soils and limited rainfall and has a flexible harvesting schedule (Ceballos et al., 2004; El‐Sharkawy, 1993; FAO & IFAD, 2000; Kizito, Chiwona‐Karltun, Egwang, Fregene, & Wasterbergh, 2007; Nweke et al., 2002). These attributes largely explain its wide‐scale adoption and cultivation across the continent.

Cassava breeding has led to significant genetic improvement for productivity traits such as fresh root yield and dry matter content (Kawano, 2003; Kawuki et al., 2011), but less so for quality traits (Ceballos et al., 2004; Lebot, 2009). Farmers in sub‐Saharan Africa are the main consumers of the cassava they produce, and they often grow multiple locally adapted varieties or elite varieties that meet specific end‐user traits including processing and cooking qualities (Alene, Khataza, Chibwana, Ntawuruhunga, & Moyo, 2013; Teeken et al., 2018; Tumuhimbise, Melis, Shanahan, & Kawuki, 2012). Previous studies have reported low adoption rates for varieties that do not meet the needs and preferences of end users (Afolami, Obayelu, & Vaughan, 2015; Alene et al., 2013). Breeding programs must therefore prioritize end‐user trait preferences to increase adoption and consequently breeding impact (Bechoff et al., 2018; Nakabonge, Samukoya, & Baguma, 2018).

In Uganda, a census of agriculture revealed that cassava is cultivated in 96.2% of the districts and that cassava is the second most important food crop after bananas (UBOS, 2010). A recent study conducted by Nakabonge et al. (2018) reported that Ugandan farmers grow cassava mainly for home food consumption and/or sale, and that different varieties are preferred for certain traits. Some of these important traits include cooking quality, storability in the soil, texture of boiled roots, and early maturity (Bechoff et al., 2018; Nakabonge et al., 2018; Tumuhimbise et al., 2012). Despite the importance of cassava in Uganda, little is known about the genetic identity and diversity of varieties currently being grown in farmers’ fields. Turyagyenda et al. (2012) studied the diversity within Ugandan farmer‐preferred varieties; however, their study was limited by a small number of individuals (51 farmer‐varieties and 15 elite accessions) and used only 26 simple sequence repeat markers. Thus, there is a need to further explore the genetic diversity of the cassava varieties grown by Ugandan smallholder farmers and determine their relationships to breeding populations using a larger number of samples and a dense set of genome‐wide markers in order to better draw inferences about the varieties grown by farmers and the breeding germplasm.

Achieving genetic gain through artificial selection requires that (a) adequate genetic variation for the trait of interest is available, (b) the trait of interest is heritable, and (c) the trait can be efficiently and effectively assessed to enable selection decisions (Falconer & Mackay, 2009). This requires a deep understanding of varieties that are currently being grown, an aspect that can be captured through quantitative assessment of their genotypic and phenotypic relationships and comparisons with elite breeding materials (Acquaah, 2012; Alene et al., 2013). Neither morphological descriptors nor variety names reported by farmers can provide unambiguous varietal identification (de Leon, Jannink, Edwards, & Kaeppler, 2016; Kizito et al., 2007; Nakabonge et al., 2018; Nduwumuremyi, Melis, Shanahan, & Theodore, 2017; Rabbi et al., 2015), a situation that can complicate excursions aimed at collecting germplasm for conservation and/or breeding purposes. In contrast, genetic markers offer robust and objective means of variety identification, which has been demonstrated for cassava farmer varieties in Ghana using genotyping‐by‐sequencing (GBS) (Rabbi et al., 2015).

To enhance cassava breeding efforts in Uganda, the objectives of this study were to assess the structure of genetic relationships among cassava cultivated in the four major cassava growing districts and to compare the genetic diversity in these farmer‐cultivated cassava to the diversity in a collection of breeding lines. To this end, we conducted a genetic survey of 547 cassava cultivated in smallholder farms in Uganda and compared these genotypes to those of 349 breeding lines from the cassava breeding program at the National Crops Resources Research Institute (NaCRRI), Namulonge, Uganda.

## 2 MATERIALS AND METHODS

### 2.1 Study sites and collection of leaf samples

This study was conducted in four districts (Apac, Arua, Kibaale, and Masindi) in Uganda (Supplemental Figure S1). These districts were selected because they are associated with high cassava production and consumption (UBOS, 2010). Also, these districts experience low prevalence of cassava mosaic and cassava brown streak disease, caused by *Cassava mosaic virus* and *Cassava brown streak virus*, respectively (Alicai et al., 2007). We conducted a survey to capture cassava trait preferences within two randomly selected villages per district. Within each village, we selected 24 smallholder cassava farmers (stratified by age and sex) using simple random sampling to participate in the study. In total, 192 farmers participated in the study from the four districts. The study plan and consent forms to engage human participants in the study were reviewed and approved by the Institutional Review Board (IRB) of Cornell University (IRB ID 1502005316). The study only commenced upon farmers granting us permission. We employed an interview guide to collect data on farming practices, cassava varieties cultivated, their names, and traits liked and disliked by farmers. In addition, we sampled three to four apical leaves from each variety the farmer was cultivating and preserved them in silica gel for genotyping (Girma et al., 2017). As noted above, we collected data from each farmer on the characteristics of the varieties they were cultivating that they preferred. We therefore refer to farmer‐cultivated varieties as farmer‐preferred synonymously. In total, this resulted in a collection of 556 samples from farmer varieties.

### 2.2 DNA extraction and genotyping

Genomic DNA was isolated from each of the collected leaf tissue samples; extraction was undertaken at NaCRRI, Namulonge, Uganda, using the method described by Dellaporta, Wood, and Hicks (1983). The DNA samples were shipped to the Cornell Biotechnology Resource Center, where they were analyzed using the GBS protocol of Elshire et al. (2011) with the *Ape*KI restriction enzyme following Hamblin and Rabbi (2014). Genotype discovery and calling was done jointly on 1,530 samples: in addition to the 556 new samples collected for this study, 624 samples described by Iragaba et al. (2019) were included as well as a random set of 350 samples that were selected from a diverse panel of breeding lines at NaCRRI (Kayondo et al., 2018). The single‐end raw reads of 150 bp were processed through the TASSEL GBS v2 production pipeline (Glaubitz et al., 2014). Genotype calls were allowed only when a minimum of two reads were present in a given sample. This process generated 470,413 single nucleotide polymorphisms (SNPs) on 1,530 samples (Supplemental Figure S2). Sites with more than two alleles, extreme deviation from Hardy–Weinberg equilibrium (χ^2^ > 20), and loci with >80% missing data were removed (Chan, Hamblin, & Jannink, 2016). We also removed samples that had >80% missing data. After this filtering, there were a total of 287,952 SNPs scored on 1,519 samples. The remaining SNP loci with missing genotypes were imputed with Beagle version 4.0 (Browning & Browning, 2009). Thereafter, we obtained a subset of 968 samples from the above 1,519 samples. The selected subset consisted of 547 farmer varieties (nine of the initial 556 samples had >80% missing data, and thus these nine were removed prior to imputation), 349 NaCRRI breeding lines, and 72 biological replicates that were the checks used in the study by Iragaba et al. (2019). These 72 biological replicates consisted of five genotypes: three released varieties, UG110017 (NAROCASS 1), UG110004 (NASE 4), and UG110014 (NASE 14); a common breeding line UG110015 (TME‐14); and a landrace, UGL15228 (Lugigana). These five genotypes had 17, 12, 19, 18, and 6 biological replicates, respectively. The bioinformatic and statistical analysis workflow is depicted in Supplemental Figure S2 for clarity.

### 2.3 Statistical analyses

Our principal objective was to determine the number of unique varieties grown in Uganda and their relative abundances. Accordingly, using the SNP marker data, we determined a threshold of genetic similarity above which differences among samples were indistinguishable, as outlined in previous studies (Myles et al., 2011; Rabbi et al., 2015). This was done using SNP data of the five genotypes that had multiple biological replicates. We used PLINK v1.90 (Purcell et al., 2007) to compute the pairwise identity‐by‐state (IBS) similarities between the replicated samples. We then used the *dist* function in the *stats* R package (v3.5.1; R Core Team, 2018) to convert the IBS matrix to a dissimilarity structure. From the distance matrix, we used the *hclust* R package to conduct Ward's hierarchical clustering.

Based on the clustering results, we determined a threshold of Ward's distance that could separate biologically replicated samples into distinct clonal groups of genotypes. The chosen threshold was subsequently used in downstream analyses to declare which varieties were distinct. The selected threshold was applied to a distance matrix of a dataset including 547 samples from farmer‐varieties, 349 breeding lines, and 72 biological replicates using the *cutree* function in the *stats* R package. To reduce redundancy of multiple samples in the same clonal group, after clustering, we subsetted each clonal group of samples such that it was represented only with a single randomly chosen sample per variety (clonal group) per district of sample origin. From this point onwards, we refer to this collection of representative samples as the set of unique varieties in each district.

As a complement to our hierarchical clustering approach to identify genetically unique varieties from the 547 samples collected from farmer varieties, we ran the ADMIXTURE model (Alexander & Lange, 2011; Alexander, Novembre, & Lange, 2009). As recommended by Alexander et al. (2009), we first filtered our dataset to obtain a SNP marker set that was mostly in linkage equilibrium using PLINK *–indep‐pairwise* with a window size of 50, step size of 10, pairwise linkage disequilibrium (LD) *r* ^2^ threshold of .3, and minor allele frequency (MAF) < .01. With the LD‐pruned dataset (119,714 SNPs), we ran the ADMIXTURE program with the ancestral population number (*K*) varying from 1 to 18 to determine the optimal *K* based on the lowest program‐reported, fivefold cross‐validation error rate. The ADMIXTURE results for the optimal *K* value were compared with the IBS‐based set of genetically unique varieties. We used this to verify that putative identical varieties had approximately the same ancestry proportions.

Additionally, using the IBS‐derived set of clonal groups, we examined the correspondence between farmer‐reported variety names and their genetic identities using a chord diagram generated with the *chordDiagram* function of the *circlize* R package. For ease of visualizing the plot, we considered only clonal groups that had >20 members and included farmer‐reported variety names that appeared >11 times in our dataset.

We quantified the overall level of genetic differentiation between districts using the fixation index (*F*_ST_) as implemented in *vcftools* (Danecek et al., 2011; Weir & Cockerham, 1984). We computed between‐district *F*_ST_ using the set of samples we described above in which each clonal group (unique variety) is represented by one sample per district in which that variety was found. Prior to *F*_ST_ computation, we removed SNPs with MAF < .01. We also used principal component analysis (PCA, *prcomp* function in R with center and scale set to TRUE) to reduce patterns of genetic relatedness in our dataset to a few dimensions that could be visually examined. Before any PCA analysis, we filtered SNPs with MAF < .01 and also removed monomorphic SNPs. In order to observe trends in diversity across the genome, we used the *vcftools* function *–window‐pi* (Danecek et al., 2011) to compute the nucleotide diversity (π) per 0.5‐Mb window for the unique set of varieties per district.

After preliminary analyses of our dataset, and given a recent study indicating the prevalence in modern cassava of large introgressions from the wild relative *Manihot glaziovii* Müll. Arg. (Wolfe et al., 2019), from our samples, we extracted the dosage of *M. glaziovii* introgression diagnostic alleles at 31,642 diagnostic markers described in Wolfe et al. (2019) (Supplemental Table 1 from that paper). We computed the proportion of *Manihot glaziovii* alleles per sample across the set of introgression diagnostic markers observed in our dataset both genome‐wide and in two focal regions described in Wolfe et al. (2019), chromosome 1 from 25 Mb to the end, and chromosome 4 from 5–25 Mb.

Lastly, to explore the relationship between varieties cultivated by farmers and the NaCRRI breeding lines, we conducted another PCA and computed *F*_ST_ and nucleotide diversity values between the farmer varieties and breeding lines. For these analyses, we used a random sample of unique varieties per district to represent the farmer varieties and all the 349 breeding lines. The nucleotide diversity per 0.5‐Mb window and *F*_ST_ were computed in *vcftools* using procedures described above. Thereafter, we plotted the distribution of the ratio of nucleotide diversity per 0.5‐Mb window of breeding lines to farmer varieties.

### 2.4 Data availability

The imputed SNP genotypic data obtained from 968 samples used in this study are available on Cassavabase website https://cassavabase.org/breeders_toolbox/protocol/6) or through its FTP (file transfer protocol; ftp://ftp.cassavabase.org/manuscripts/Iragaba_et_al_2019_diversity/Genotype_infos/) in a file named Iragaba GBS.vcf.gz.

## 3 RESULTS

### 3.1 Number of unique varieties grown in Uganda and their relative abundances

In total, we successfully genotyped 547 leaf samples collected from different cassava plants grown by 192 smallholder farmers. Collectively, this translated to 156, 139, 137, and 115 samples that were sourced from farmers’ fields in Kibaale, Arua, Masindi, and Apac, respectively (Table 1, Supplemental Table S1). Based on varietal names assigned by farmers, we recorded an average of three varieties cultivated per farmer in Arua, Kibaale, and Masindi districts, and two in Apac. Overall, some farmers were growing as few as one and as many as six varieties.

**TABLE 1:** Summary of leaf samples collected from cassava plants grown by smallholder farmers within four districts in Uganda and the number of unique varieties per household

District	Total no. of samples collected	Avg. no. of samples per household	Total no. of unique varieties^a^	Avg. no. of unique varieties per household
Apac	115	2.4	21	2.3
Arua	139	2.8	24	2.6
Kibaale	156	3.4	41	3.3
Masindi	137	2.7	33	2.6

^a^Unique varieties determined based on identity‐by‐state distinctions.

A Ward's distance threshold of 0.075 clearly grouped biological replicates together and distinctly separated the five known genotypes—UG110017 (NAROCASS 1), UG110004 (NASE 4), UG110014 (NASE 14), UG110015 (TME‐14), and UGL15228 (Lugigana)—from each other (Supplemental Figure S3). After applying this threshold to the 547 experimental samples collected from farmers, we identified a total of 86 unique varieties. Most of these unique varieties (*n *= 65) were only found in a single district: 30 in Kibaale, 19 in Masindi, 13 in Arua, and three in Apac. Of the remaining 21 unique varieties, only three were present in all four districts, six were present in at least three districts, and 12 were present in at least two districts (Supplemental Table S2). Similar to farmer‐reported variety names, we found an average of 2.3 genetically unique varieties cultivated per farmer in Apac, whereas an average of 2.6–3.3 distinct varieties were cultivated per farmer in the other three districts (Table 1). Most of the identified unique varieties were observed less than five times (*n *= 60); only 14 varieties were observed >10 times (Supplemental Table S3).

**FIGURE 1:**
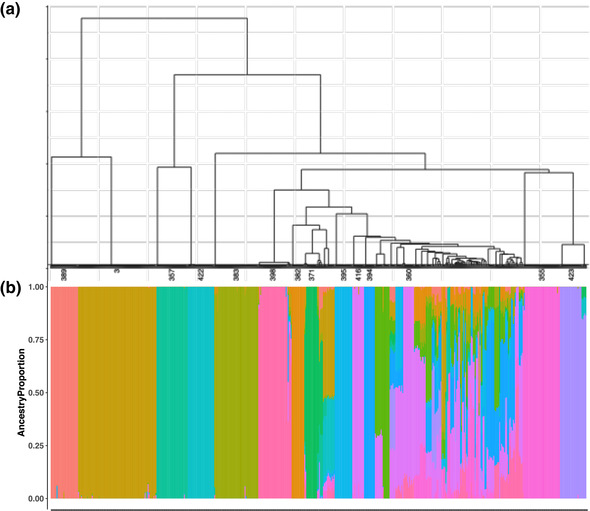
(a) Dendrogram from Ward's hierarchical clustering of identity‐by‐state (IBS) matrix with the 14 largest clonal groups labeled to show correspondence with Panel b. Population structure (based on subpopulations, *K *= 14) analysis on 547 cassava samples using ADMIXTURE. In the panel, each sample is indicated by a vertical bar partitioned in one or more colored segments, and the respective length of the bar represents the proportion of the individual's genome ancestry in a given subpopulation. Each color represents a different ancestry subpopulation

To complement IBS results, we used ADMIXTURE analysis on the 547 samples at *K *= 14 because that had the lowest cross‐validation error rate (Supplemental Figure S4). We observed that these 547 samples with the same proportion of ancestry were almost always identified to be in the same clonal group derived from the Ward's threshold (Figure 1, Supplemental Table S1). For example, all samples (*n = *80) in Clonal Group 3 had ∼100% of their proportion derived from Ancestry 11. Similarly, all samples (*n = *38) belonging to Clonal Group 355 were entirely derived from Ancestry 8 (Supplemental Table S1).

### 3.2 Genetic relationship among cassava varieties cultivated in different districts in Uganda

**FIGURE 2:**
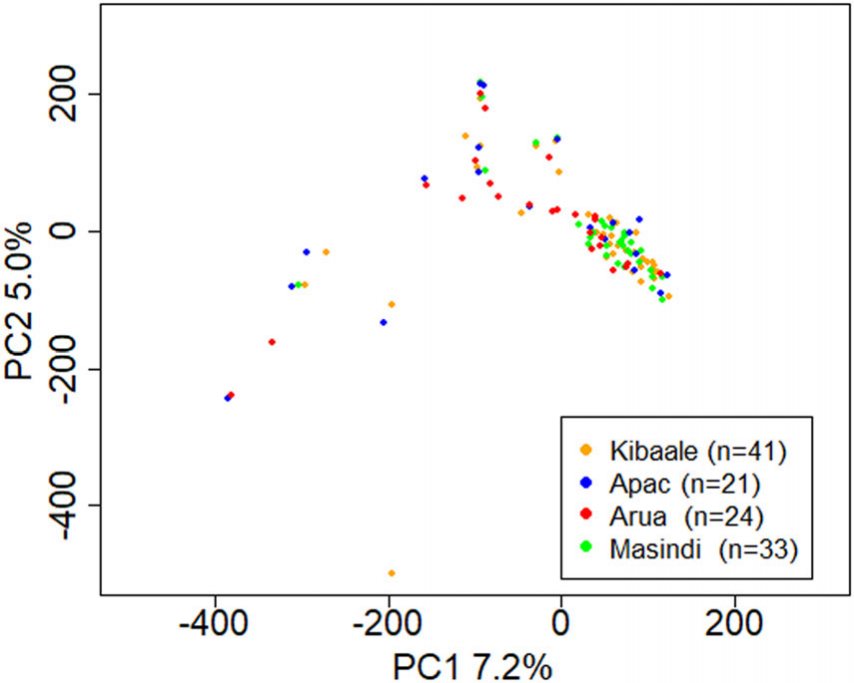
Principal Components (PC) 1 and 2 based on 189,851 genome‐wide single nucleotide polymorphisms (SNPs) scored on 119 genetically unique cassava varieties randomly selected from the clonal groups in each district after correction for multiple samples within the same clonal group (if a unique variety was represented in two or more districts, it had one entry for each of the districts) colored by district from where the samples were collected indicated minimal clustering in relation to the source of the sample. One of the samples from Kibaale district was an outlier (orange dot at the extreme low end of PC2)

The genetic divergence between the 86 unique cassava varieties cultivated in the four districts was low, with *F*_ST _< .05 for all pairwise comparisons (Table 2). Additionally, results from PCA indicated no clear clustering pattern of varieties based on their location (Figure 2). The percentage of variance explained by each of the principal components (PCs) was relatively low (Supplemental Figure S5). Furthermore, the average nucleotide diversity among farmer varieties was highest in Apac (1.06 × 10^−4^) and lowest in Masindi (1.01 × 10^−4^) (Table 3).

**TABLE 2:** Fixation index (*F*_ST_) estimates among cassava varieties cultivated by smallholder farmers in four districts of Uganda

District	Kibaale (*n *= 41)	Masindi (*n *= 33)	Arua (*n *= 24)	Apac (*n *= 21)
Kibaale	–			
Masindi	.002096	–		
Arua	.007788	.006847	–	
Apac	.002616	.002748	−.003265	–

We used a pruned dataset with only 119 samples representing distinct farmer‐varieties randomly selected from within each district

**TABLE 3:** Average nucleotide diversity (π) per chromosome for unique cassava varieties grown by smallholder farmers within four districts in Uganda

Chromosome	Apac π (*n *= 21)^a^	Arua π (*n *= 24)^a^	Kibaale π (*n *= 41)^a^	Masindi π (*n *= 33)^a^
1	0.0001574	0.0001479	0.0001445	0.0001379
2	0.0001162	0.0001154	0.0001144	0.0001107
3	0.0001106	0.0001105	0.0001091	0.0001072
4	0.0001223	0.0001184	0.0001094	0.0001099
5	0.0001041	0.0001044	0.0001040	0.0001035
6	0.0001149	0.0001129	0.0001120	0.0001089
7	0.0000849	0.0000847	0.0000860	0.0000837
8	0.0000861	0.0000846	0.0000815	0.0000814
9	0.0000919	0.0000942	0.0000908	0.0000904
10	0.0001158	0.0001158	0.0001099	0.0001095
11	0.0001208	0.0001199	0.0001147	0.0001152
12	0.0000808	0.0000804	0.0000780	0.0000784
13	0.0000832	0.0000841	0.0000833	0.0000818
14	0.0001307	0.0001318	0.0001286	0.0001238
15	0.0001225	0.0001230	0.0001206	0.0001196
16	0.0000828	0.0000831	0.0000775	0.0000771
17	0.0000921	0.0000945	0.0000890	0.0000874
18	0.0000906	0.0000906	0.0000874	0.0000887
Avg.	0.0001060	0.0001053	0.0001023	0.0001008

^a^ *n* represents the number of unique farmer varieties per district used in the computation of nucleotide diversity.

### 3.3 Correspondence between variety names reported by the farmers and their genetic identity

**FIGURE 3:**
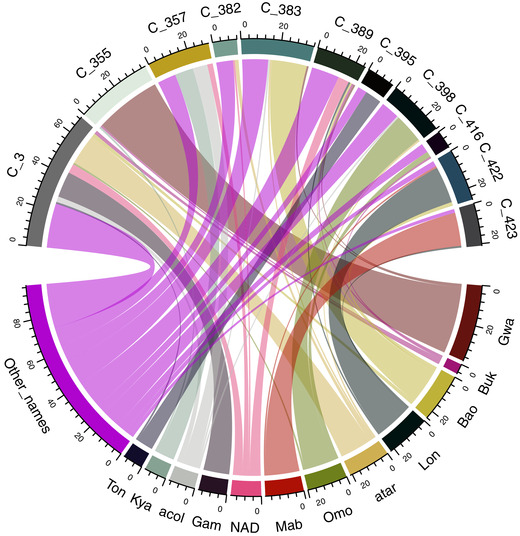
Correspondence between genetic identity of 11 clonal groups, labeled C_3 to C_423 (upper semicircle) and most common variety names given by farmers (lower semicircle). Clonal groups with >11 members were considered, and farmer‐reported variety names that were mentioned >11 times in total are presented in this plot. The numbers on the axis represents either the number of members (count) in a given clonal group or the number of times a given variety name was mentioned during the surveys. The label for “Other_names” represents all other variety names that had a count <11 times. The abbreviations for 11 variety names: Ton, Tonguda; Kya, Kyawada; acol, Gamente acol; Gam, Gamente; NAD, NAADs; Mab, Mabulu; Omo, Omoo; atar, Gamente atar; Lon, Longe; Buk, Bukalasa; Gwa, Gwalanda

Based on farmer‐reported naming, 156 unique varieties were reported (Supplemental Table S4). Variety names ‘Gwalanda’, ‘Bukalasa’, ‘Bao’, and ‘Longe’ were the most common, accounting for 22.5% of the samples collected (Supplemental Table S4). Overall, farmer‐reported variety names did not reliably correspond to genetically unique varieties as empirically revealed by SNP markers (Figure 3). For example, the largest clonal group (C_3), which was observed 80 times, included members that had up to 32 different variety names assigned by farmers (Figure 3, Supplemental Table S3). However, there were instances when almost all farmer‐reported variety names agreed within a given clonal group. For instance, 80% of the samples referred to as ‘Gwalanda’ had the same genetic identity (C_355) derived from IBS similarities (Figure 3, Supplemental Table S3).

**FIGURE 4:**
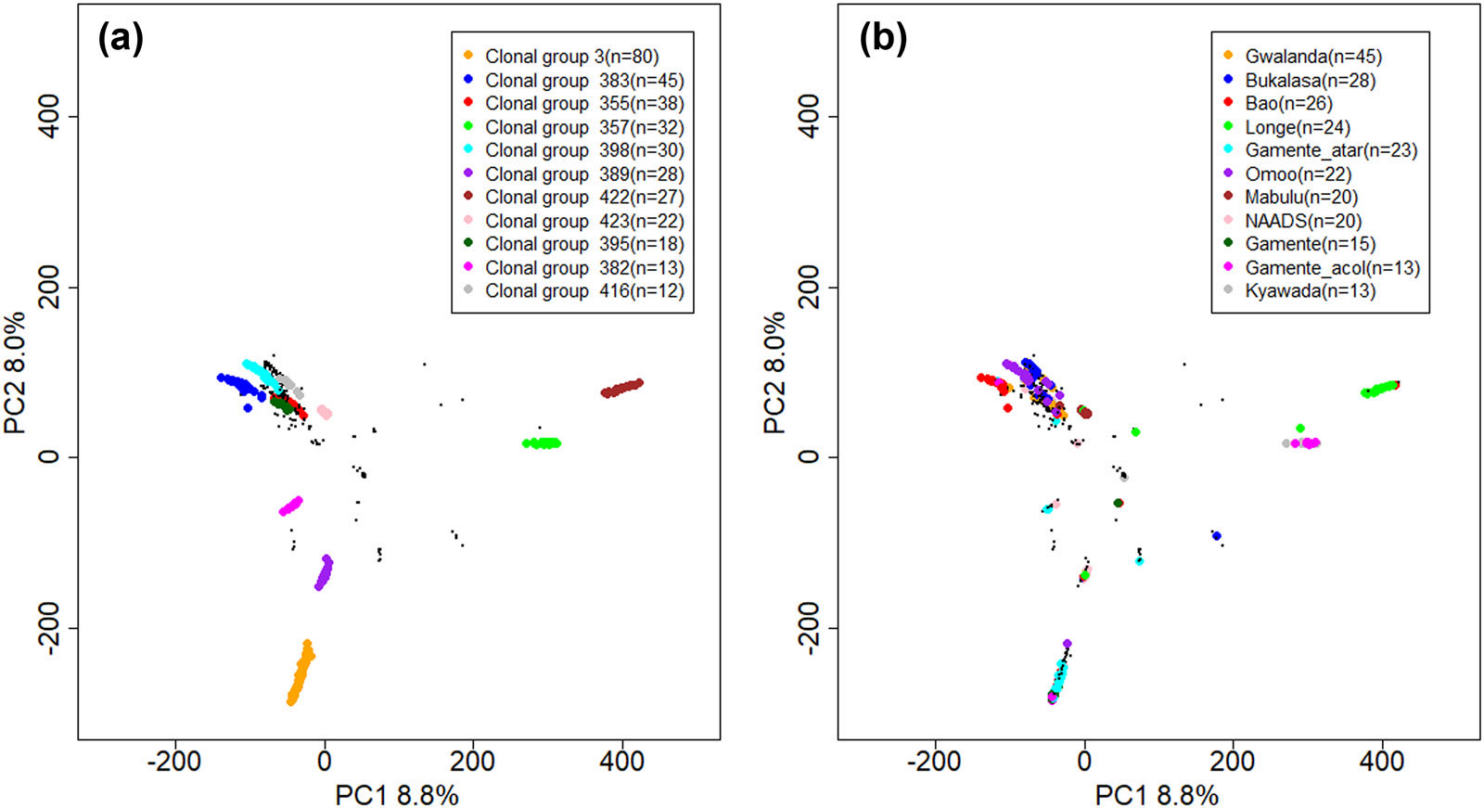
Principal Components (PC) 1 and 2 based on 190,556 genome‐wide single nucleotide polymorphisms (SNPs) scored on 547 samples colored by the (a) 11 most common clonal groups from identity‐by‐state (IBS)‐based distinctions and (b) names of the 11 predominant cassava varieties reported by farmers

To further visualize how genetically identical varieties derived from IBS similarities related to the respective farmer‐reported variety names, the first two genetic PCs were colored based on the most predominant (at least *n *= 12) clonal groups (Figure 4a) and the most predominant (at least *n *= 13) variety names reported by farmers during the survey (Figure 4b). The structuring pattern in the PCA plots indicated that members of the same clonal group grouped together as expected. However, when the same plot is colored based on variety names given by farmers, members with similar names often did not group together (Figure 4). Taken together, these results confirmed that a number of genetically unique varieties had multiple names reported by farmers. This phenomenon was observed both within and between districts (Supplemental Table S1).

### 3.4 Genetic relationships among farmer‐grown cassava varieties and breeding lines in Uganda

**FIGURE 5:**
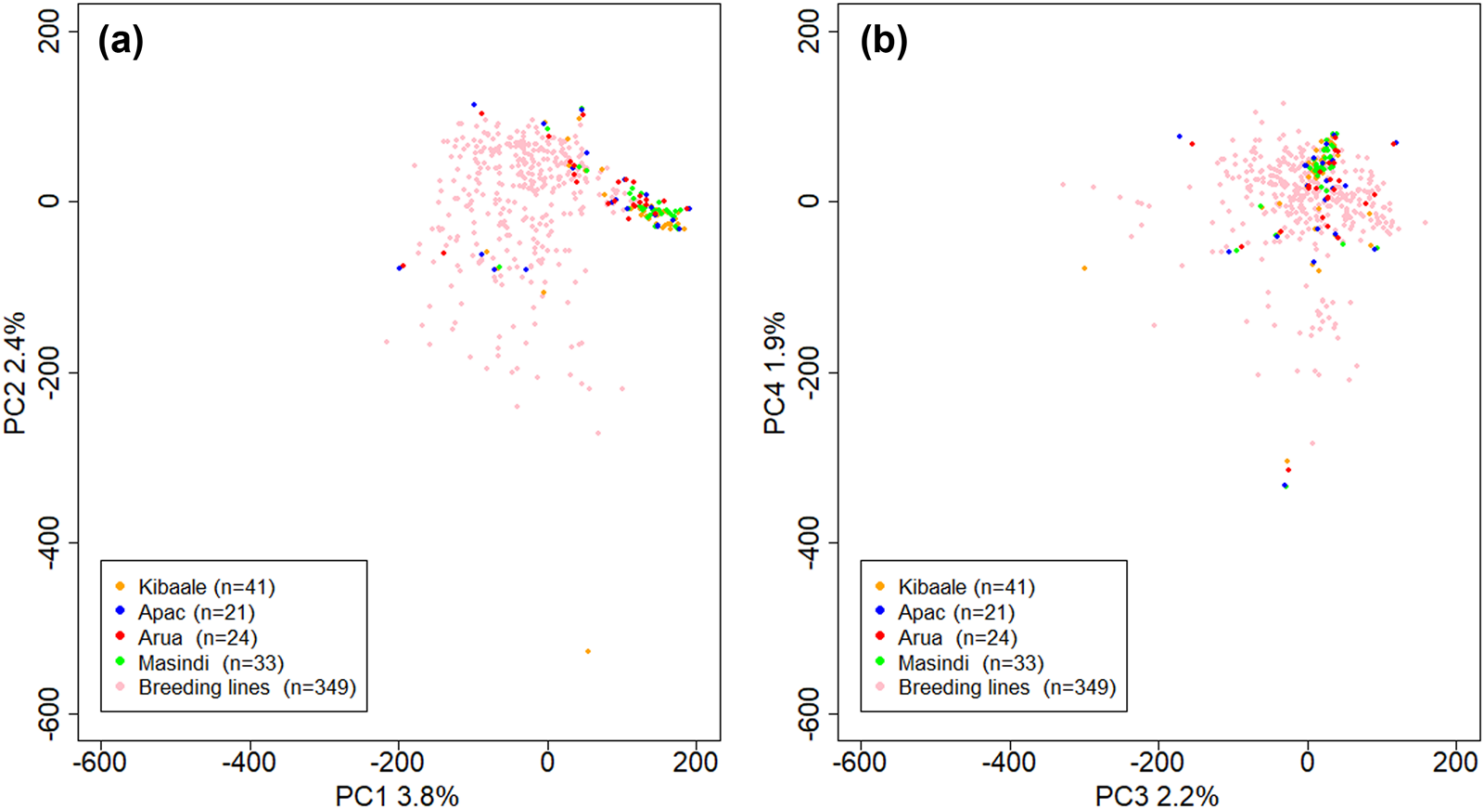
(a) Principal Components (PC) 1 and 2 and (b) 3 and 4 based on 349 breeding lines and 119 unique farmers’ varieties scored on 201,117 single nucleotide polymorphisms (SNPs). The plots show patterns of structure between cassava varieties cultivated by smallholder farmers in four districts (Kibaale, Apac, Arua, and Masindi) in Uganda and the cassava breeding lines at the National Crops Resources Research Institute (NaCRRI), Uganda. Only unique varieties per district are used for the farmer varieties. One of the samples from the Kibaale district was an outlier (orange dot at the extreme low end of PC2 in Panel a)

**FIGURE 6:**
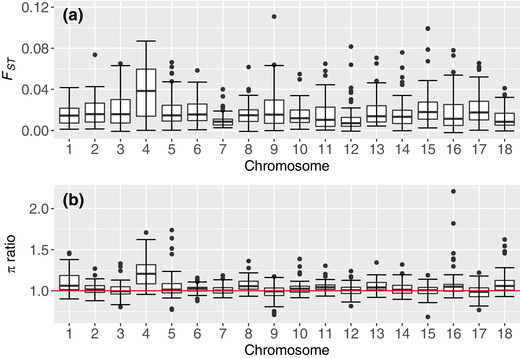
(a) Mean fixation index (*F*_ST_) per 0.5‐Mb window between cassava breeding lines at the National Crops Resources Research Institute (NaCRRI) and cassava varieties grown by smallholder farmers in Uganda. (b) Mean nucleotide diversity (π) ratio per 0.5‐Mb window of breeding lines to farmer varieties. The red line in Panel b is the threshold above which the mean π in breeding lines is higher than that in farmer varieties, and below which the mean nucleotide diversity in breeding lines is less than that in farmer varieties

Our results based on IBS indicate that all cassava varieties cultivated by farmers in Apac, Arua, Kibaale, and Masindi districts are not clones of the 349 breeding lines sourced from NaCRRI (Supplemental Table S5). We also conducted a PCA to visualize how farmers’ varieties related to breeding lines. In the genetic space described by the first four PCs, the farmer varieties are largely a subset of the breeding lines (Figure 5). That is, though we found no clonal relationships, the farmer varieties appear to have close relatives among the breeding lines. The percentage of variance explained by each of the PCs was low (<4%) (Supplemental Fig. S5). The *F*_ST_ indicated low genetic differentiation (.020) between farmers’ varieties and breeding lines. The mean nucleotide diversity among breeding lines (1.08 × 10^−4^) was higher than that of farmer varieties (1.03 × 10^−4^) (Figure 6). The mean level of homozygosity was very similar between the breeding lines (69.95%) and farmer varieties (70.01%), although a few breeding lines had particularly high levels of inbreeding (Supplemental Table S5, Supplemental Figure S6). The highest ratio of nucleotide diversity of breeding lines to farmer varieties was observed on chromosomes 4, 18, and 1, respectively, whereas the lowest ratio was observed on chromosome 9.

We also detected *M. glaziovii* introgressions in both the farmer and breeding lines, based on available introgression diagnostic markers (Wolfe et al., 2019). We detected introgressions, as expected, mostly on chromosomes 1 and 4, with a per‐individual genome‐wide frequency of on average 0.047 among breeding lines and 0.03 among the farmer varieties (Supplemental Table S5, Supplemental Figure S7).

## 4 DISCUSSION

Comparing genetic relationships among varieties adopted by farmers with those of breeding lines is important in developing new varieties that best meet the needs and preferences of the end users. This study revealed that despite the low (*F*_ST_ < .05) genetic differentiation among cassava varieties grown in different districts of Uganda, the varieties cultivated across different districts are often distinct genetically. Of the 547 samples collected from Ugandan farmers, there were 86 genetically unique varieties. Of these unique varieties, most of them were cultivated in only one single district (30 in Kibaale, 19 in Masindi, 14 in Arua, and three in Apac), whereas only three were cultivated across all the four districts. However, these unique varieties are likely to be close relatives, given the observed levels of genetic differentiation between districts.

In our study, we found, in agreement with a recent study in Ghana (Rabbi et al., 2015), that most smallholder farms cultivate two or more cassava varieties in the same field in order to meet the diverse needs of farmers and end users (Nweke et al., 2002). Consequently, the different unique varieties could be serving different purposes both for the farmer (risk aversion, in case one variety or market fails) and for the consumer (processing, fresh consumption) (Nakabonge et al., 2018). For instance, during the interviews prior to leaf sample collection, some farmers mentioned that certain varieties were used as a source of food for the household members, whereas other varieties were largely for income generation. The genetic differentiation we observed among farming districts in Uganda (mean *F*_ST_ = .003) is similar to that observed between the two breeding programs based within Nigeria (Wolfe et al., 2017; *F*_ST_ = .008) and lower than observed levels of differentiation between, for example, East and West Africa (Ramu et al., 2017; Wolfe et al., 2017), which range from .01 to .05. The levels of genetic differentiation observed in cassava populations may be due to the common practice of exchanging planting materials between neighboring farmers, friends, and relatives (Mtunguja, Laswai, Muzanila, & Ndunguru, 2014). In addition, cassava is known to have a high outcrossing rate in the field (da Silva, Bandel, & Martins, 2003), and recombinant seed can establish in farmers’ fields, be erroneously propagated, and lead to new varieties that are closely related to what is already in production (Duputie, Deletre, Granville de, & Mickey, 2009; Fregene et al., 2003). Thus, continued gene flow within the continent is likely to be a significant factor in the limited population structure that has been observed.

In this study, we showed that farmer‐reported variety names were not consistent with the genotype information. For example, the variety names Akena, Bao, Bukalasa, Gamente, Gotta, Kibaho, Mukuma, NAADS, and Olam that were assigned by the farmers were classified under the same genetic identity (Clonal Group 3). The implication of this result is that breeders should not solely rely on the farmer‐given variety name in variety identification studies. This is in agreement with previous studies, which have also reported a large discrepancy between genetically unique varieties and the variety names assigned by farmers (Bredeson et al., 2016; Rabbi et al., 2015). Indeed, most farmers obtain cassava varieties from their neighboring farmers, relatives, and friends (Nweke et al., 2002; Teeken et al., 2018). The inconsistency between genotype and variety names is thus attributable to the lack of a regulated seed system with the ability to maintain genetic fidelity relative to germplasm names. A previous study revealed that naming of cassava varieties is subjective and may depend on many factors, such as the place of origin, maturity period, taste, morphology, yield, marketability, and resilience (Kizito et al., 2007; Nakabonge et al., 2017). Indeed, we observed that some variety names (e.g., Bukalasa) refer to the place or source of its origin while others refer to phenotypes. For example, Gamente‐acol may have been sourced from the government, as Gamente is the local language name for the government, and the last part of the name separated by a hyphen (acol) is derived from the color of stems that are mostly dark (acol means dark in the local language). Our results indicate that variety name alone is not reliable and should not be used to define unique cassava varieties in studies of adoption by local farming communities in Uganda or for the collection of farmer varieties to be used in breeding. In a few scenarios, the samples with a similar variety name belonged to the same clonal group (e.g., of the samples that were named as Gwalanda, 80% of them belonged to the same clonal group; Figure 3). All the samples named Gwalanda were collected from Kibaale district, and one of the possibilities for the observed variation in naming pattern was due to the distinct morphological characteristics upon which the variety name was derived.

All breeding lines that we analyzed were genetically different from varieties cultivated by farmers, though we did not comprehensively sample all breeding lines and those we analyzed are known not to have yet been released to the farmers. The differentiation we observed between breeding lines and farmers’ varieties was similar to the level observed between breeding programs in Nigeria (Wolfe et al., 2017) and implies that both populations share a large number of alleles. In a previous study of Uganda farmer‐preferred varieties, Turyagyenda et al. (2012) also found that genetic distance between landraces and breeding lines was small. Indeed, the genetic variability among breeding lines along the first four PCs (Figure 5) was greater than among the farmers’ varieties, matching the observation that the breeding population is slightly more diverse (Figure 6, Supplemental Table S5) and similarly homozygous (Supplemental Figure S7). There was only one farmer variety that was notably distinct (along PC2).

Chromosomes 1 and 4 appeared notably more diverse among the breeding lines compared with farmer varieties (Figure 6). Based in part on this result, as well as those of Bredeson et al. (2016), we suspected that some of the farmer accessions might contain introgression segments from the wild relative *M. glaziovii*. Recently, Wolfe et al. (2019) revealed that the introgressions on chromosomes 1 and 4 are common in breeding germplasm, and also present (but less common) in landraces. Based on introgression diagnostic markers, we found that the same was true of the difference between breeding lines and the farmer varieties we sampled in Uganda (Supplemental Table S5, Supplemental Figure S7; Wolfe et al., 2019). Interestingly, the farmer variety mentioned above as an outlier on PC2 (Figures 2 and 5, orange dot at the extreme low end of PC2) appears to be an F_1_ (39.6% introgression diagnostic alleles, mostly in the heterozygous state) hybrid between an *M. glaziovii* and an *M. esculenta* parent. The passport data we collected from the farmer indicate that it had very bitter roots and leaves relative to other cassava varieties and that it was mainly used as a border row to deter thieves and animals from the main crop. This kind of information highlights the multiple functions of cassava varieties grown by farmers and the value of genetic surveys of farmer‐preferred varieties.

Overall, findings from this study indicate that most smallholder farmers cultivated more than one type of variety, a result comparable with what was observed in Ghana (Rabbi et al., 2015). Additionally, similar to what was reported by Rabbi et al. (2015), findings from this study revealed that SNP markers provided more reliable results for variety identification as opposed to the names of varieties provided by the farmers. Unlike what was done by Rabbi et al. (2015), our study further investigates the relationships between the varieties grown by smallholder farmers and the genotypes being used in the national breeding program in Uganda.

## 5 CONCLUSION

In this study, cassava leaf samples collected from 547 different cultivated plants grown by smallholder farmers within four districts in Uganda were genotyped with the major objective of understanding the genetic relationship among varieties grown by the farmers. We also explored the genetic relationship between these surveyed farmer varieties and breeding lines used at NaCRRI. We found that most farmers in Uganda grow two or three distinct cassava varieties and that each sampled district in Uganda contains several varieties not grown in other districts. The overall level of genetic differentiation between districts is relatively low, as is the divergence between farmer and breeding populations. Despite the fact that none of the breeding lines were directly observed in farmer fields, the presence of *M. glaziovii* introgressions on chromosomes 1 and 4 implies ancestry with cassava breeding lines. Given the apparently similar pool of alleles in the breeding germplasm, it is likely that breeders have the raw genetic material they require to match the farmer‐preferred trait combinations necessary for adoption. Our study highlights the importance of understanding the genetic makeup of cassava currently grown by smallholder farmers and relative to that of plant breeding germplasm.

## CONFLICT OF INTEREST

The authors declare that they have no conflict of interest.

## Supporting information

SUPPORTING MATERIAL

SUPPORTING MATERIAL

SUPPORTING MATERIAL

SUPPORTING MATERIAL

SUPPORTING MATERIAL

SUPPORTING MATERIAL
