# Supplementary material for: Genomic characterization of Ugandan smallholder farmer‐preferred cassava varieties
Source: Crop Sci. 2020 May 4;60(3):1450–61. doi: 10.1002/csc2.20152 (PMC7386927; doi:10.1002/csc2.20152)
Supplement: Supplementary file 1 — SUPPORTING MATERIAL [file CSC2-60-1450-s001.docx]

# SUPPLEMENTARY FIGURES: Genomic characterization of Ugandan smallholder farmer-preferred cassava (*Manihot esculenta* Crantz) varieties

Paula Iragaba^1^, Robert S. Kawuki^2^, Guillaume Bauchet^3^, Punna Ramu^4^, Hale A. Tufan^1,5^, Elizabeth D. Earle^1^, Michael A. Gore^1^ and Marnin Wolfe^1^

#

#
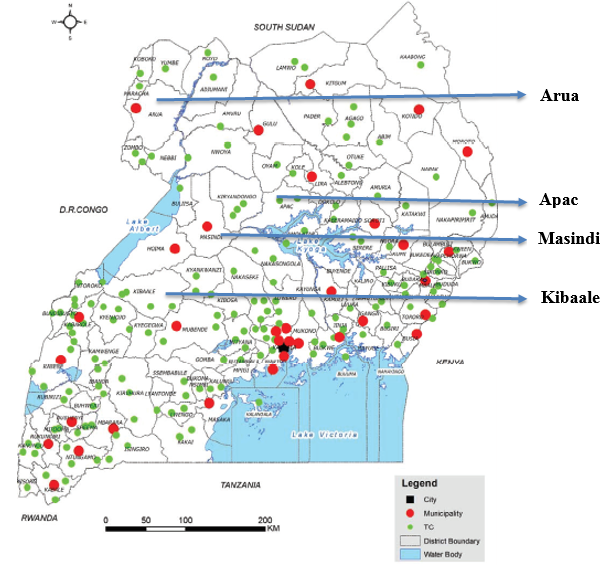


Source: UBOS (2016)

Supplementary Fig. 1. Map of Uganda highlighting four districts from where cassava leaf samples were collected. The word “TC” in the legend represents town council.


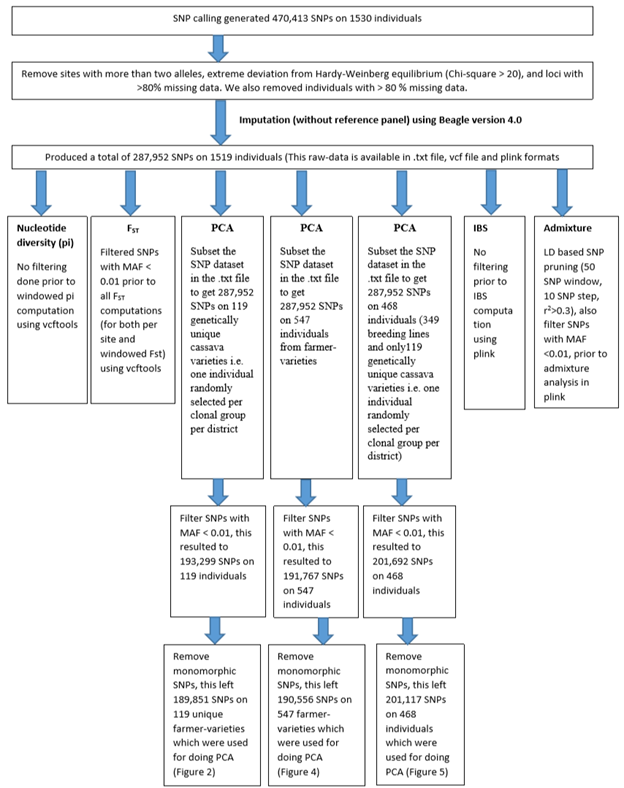


Supplementary Fig. 2. Flowchart indicating the processing pipeline that was done on the genotype data used for the statistical analyses of this paper.


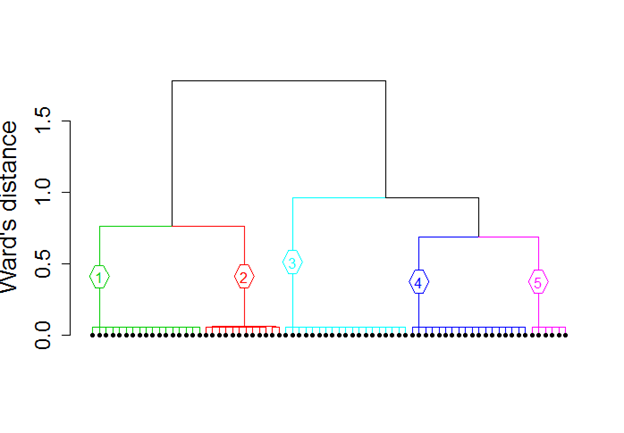


Supplementary Fig. 3. Dendrogram showing clustering of biological replicated samples of five known genotypes: 1) UG110017 (green), 2) UG110004 (red), 3) UG110014 (cyan), 4) UG110015 (blue), and 5) UGL15228 (purple), that were used to declare the threshold for identifying unique varieties from samples collected from cassava varieties grown by smallholder farmers in Uganda. The nodes in the figure represent the different biological replicates.


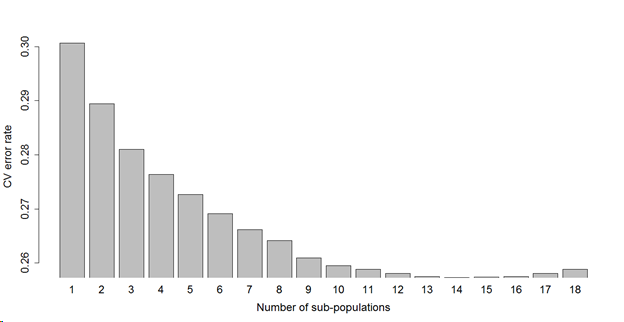


Supplementary Fig. 4. Estimated five-fold cross-validation (CV) error rate of possible sub-populations (*K*) from *K* = 1 to 18 for population structure analysis using ADMIXTURE on 547 samples collected from cassava varieties grown by smallholder farmers in Uganda. The lowest CV error rate was observed at *K* =14.


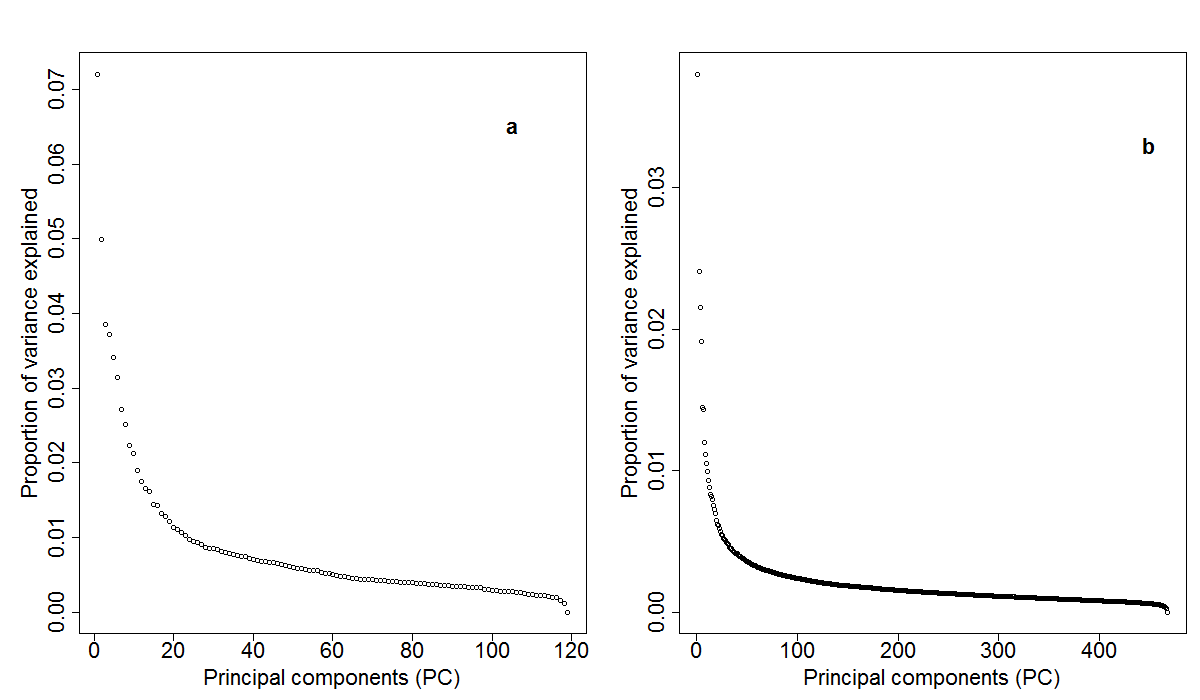


Supplementary Fig. 5. Scree plots indicating the proportion of variance explained by the principal components from PCA: a) Scree plot for the 119 unique farmer-varieties at district level b) Scree plot for the 119 unique farmer-varieties at district level and 349 breeding lines.


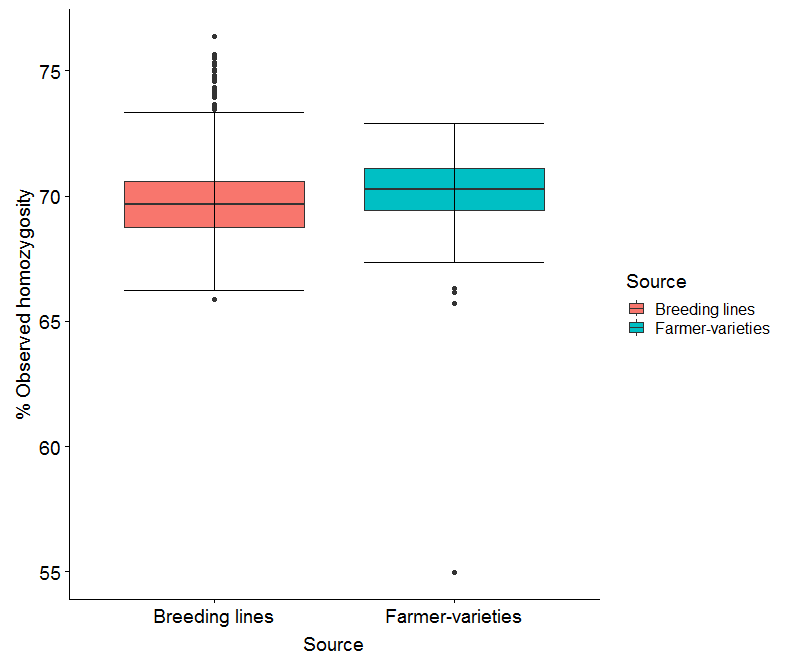


Supplementary Fig. 6. The level of observed homozygosity of the NaCRRI breeding lines and the farmer-varieties grown in Uganda. The farmer-varieties are subset of randomly selected unique individuals per clonal group per district.


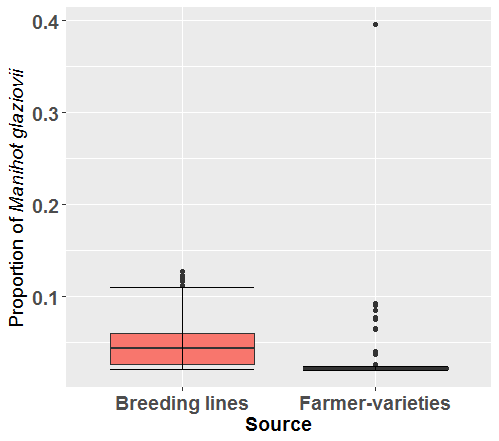


Supplementary Fig. 7. Boxplot indicating the overall proportion of *Manihot glaziovii* introgressed into the NaCRRI breeding lines and the farmer-varieties grown in Uganda. The farmer-varieties are subset of selected unique individuals per clonal group per district.
